# Factors Associated With the Rising Trend in Self‐Reported Cognitive Disability Among U.S. Adults Aged 18–39 From 2013–2024

**DOI:** 10.1002/acn3.70464

**Published:** 2026-07-05

**Authors:** Adam de Havenon, Ka‐Ho Wong, Mirriam Mananah, Christopher D. Anderson, Ada Fenick, Amy Arnsten, Arman Fesharaki‐Zadeh, James C. McPartland, Mary L. Adams, Kevin N. Sheth

**Affiliations:** ^1^ Department of Neurology Yale University New Haven Connecticut USA; ^2^ Center for Brain and Mind Health Yale University New Haven Connecticut USA; ^3^ Department of Neurology University of Utah Salt Lake City Utah USA; ^4^ Department of Population Health Sciences University of Utah Salt Lake City Utah USA; ^5^ Department of Neurology Mass General Brigham Boston Massachusetts USA; ^6^ Department of Pediatrics Yale University New Haven Connecticut USA; ^7^ Departments of Neurology and Psychiatry Yale University New Haven Connecticut USA; ^8^ Child Study Center Yale University New Haven Connecticut USA; ^9^ Retired, Connecticut Department of Public Health Hartford Connecticut USA

**Keywords:** cognitive disability, cognitive impairment, young adults

## Abstract

**Objective:**

Building on our prior Behavioral Risk Factor Surveillance System analysis identifying adults aged 18–39 as the primary driver of the national increase in self‐reported cognitive disability, we examined factors associated with this rise using 2013–2024 U.S. BRFSS data.

**Methods:**

We analyzed U.S. Behavioral Risk Factor Surveillance System data, excluding 2020 due to poor survey response, to assess trends in self‐reported cognitive disability among adults aged 18–39, excluding and including respondents with depression. Cognitive disability was defined as serious difficulty concentrating, remembering, or making decisions due to a physical, mental, or emotional condition. We compared younger adults with versus without cognitive disability across sociodemographic, socioeconomic, health, and mental health characteristics. Analyses incorporated survey weighting to yield U.S. nationally representative estimates.

**Results:**

Cognitive disability prevalence increased from 5.1% in 2013 to 9.8% in 2024 (93% increase; *p* < 0.001). Including respondents with depression, prevalence increased from 10.0% to 17.8% (16.2 million individuals). Among younger adults with cognitive disability, employment increased (55.6% to 65.1%), lack of health insurance declined (35.7% to 19.7%), smoking decreased (28.7% to 14.1%), and physical inactivity decreased (26.9% to 22.3%). Point estimates for hypertension, hyperlipidemia, asthma, diabetes, and stroke prevalence were also lower. In contrast, frequent poor mental health (≥ 14 days/month) increased from 26.6% to 33.2%. In sensitivity analyses excluding depression and any poor mental health days, prevalence still increased from 2.8% to 5.2% (*p* < 0.001).

**Interpretation:**

From 2013–2024, self‐reported cognitive disability among younger United States adults nearly doubled despite improving socioeconomic and cardiometabolic indicators, highlighting a growing burden of perceived or functional cognitive difficulty.

## 1 Introduction

Cognitive impairment affects a substantial and growing proportion of the US adult population [1, 2, 3, 4, 5, 6]. A recent analysis of Behavioral Risk Factor Surveillance System (BRFSS) data demonstrated a concerning increase in the prevalence of self‐reported “cognitive disability” from 5.3% in 2013 to 7.4% in 2023 [7]. This increase predominantly affected younger adults aged 18–39 years, in whom prevalence nearly doubled from 5.1% in 2013 to 9.7% in 2023, a 90% relative increase [3, 4]. In contrast, adults aged 70 and older showed a slight decline, from 7.3% to 6.6% [7].

The concentration of increasing cognitive disability among younger adults during critical life stages for education, career development, and family formation has important implications for long‐term workforce participation and societal support needs [8, 9, 10]. The last decade encompasses significant societal changes, including increased digital and social media consumption, a global pandemic, implementation and expansion of the Affordable Care Act, evolving attitudes toward neurodiversity, and unprecedented mental health challenges for younger adults [11, 12, 13, 14, 15].

The current study aims to comprehensively examine characteristics, socioeconomic indicators, health behaviors, and chronic conditions among adults aged 18–39 years with and without cognitive disability. We perform analyses that both exclude and include individuals ever diagnosed with depression, which is associated with frontal‐subcortical and hippocampal dysfunction and persistent memory and executive‐function deficits that could confound the analysis [16, 17, 18, 19, 20]. We also examined temporal changes in the rates of other forms of disability in BRFSS and changes in social and medical characteristics among younger adults with cognitive disability [3].

## 2 Methods

### 2.1 Study Design and Data Source

We conducted a serial cross‐sectional analysis using BRFSS data from 2013 to 2024, excluding 2020 due to pandemic‐related data collection disruptions [21]. BRFSS is an ongoing, state‐based telephone survey administered by the Centers for Disease Control and Prevention (CDC) that collects health‐related data from non‐institutionalized adults aged 18 years and older across all 50 US states, the District of Columbia, and participating US territories [21, 22, 23]. The survey uses random‐digit dialing to conduct interviews via landline and cellular telephones [21, 23].

Disability status was self‐reported using six standardized questions included in the BRFSS questionnaire, consistent with the Department of Health and Human Services (DHHS) disability classification framework (Table S1).

BRFSS classified respondents answering yes to any question as having that specific type of disability. Data on hearing disability are only available for 2016–2024. For this analysis, we first examined trends in all six self‐reported disability types and then focused on a detailed characterization of cognitive disability.

### 2.2 Standard Protocol Approvals, Registrations, and Patient Consents

Because the BRFSS dataset is fully de‐identified and publicly available, it does not require standard protocol approvals, registration, or patient consent. The data are accessible to anyone and can be freely downloaded from the CDC website [21]. Additionally, researchers may request specific datasets or documentation directly from the CDC, making BRFSS a widely accessible resource for population health research that complies with federal regulations regarding non‐human subjects research.

### 2.3 Study Population

The primary analysis excluded adults who endorsed a history of depression (“Have you ever been told you had a depressive disorder, including depression, major depression, dysthymia, or minor depression?”) [22]. By excluding adults with depression, we aimed to characterize cognitive disability that was not secondary to or confounded by the most common mood disorder known to impair cognition [20, 24, 25].

### 2.4 Variables

- *Demographics:* Age (categorized as 18–24, 25–29, 30–34, 35–39 years), sex, race/ethnicity (White, Black, American Indian/Alaskan Native [AI/AN], Asian, Hispanic, Other), Census Region (Northeast, Midwest, South, West, Territories).
- *Socioeconomic Factors:* Educational attainment (less than high school, high school graduate, some college, college graduate), household income (< $35,000, $35,000–$74,999, ≥ $75,000), employment status (employed for wages, unemployed, unable to work, student, homemaker, retired), health insurance status (insured vs. uninsured).
- *Health Behaviors:* Smoking status (never smoker, former smoker, current smoker), any alcohol use, any leisure‐time physical activity in the past 30 days (physically inactive defined as no activity).
- *Chronic Conditions:* Self‐report of ever having hypertension, hyperlipidemia, diabetes mellitus, chronic kidney disease, asthma, myocardial infarction, stroke, obesity (body mass index ≥ 30 kg/m^2^). Note that the hypertension and hyperlipidemia questions were assessed in alternating years only (2013, 2015, 2017, 2019, 2021, 2023), limiting the years available for these variables.
- *Poor Mental Health:* “Thinking about your mental health, which includes stress, depression, and problems with emotions, for how many days during the past 30 days was your mental health not good?” We used the poor mental health variable as both a continuous value and transformed the values into a nominal variable representing 0 days, 1–13, or 14 or more days a month.

### 2.5 Statistical Analysis

Baseline characteristics were compared using unweighted analyses. All subsequent analyses accounted for the complex survey design of the BRFSS, incorporating sampling weights, primary sampling units, and stratification variables to generate population‐representative estimates. Analyses were performed in Stata version 18.0 using the survey commands, with single‐unit strata centered and the subpopulation option applied to restrict analyses to relevant groups (e.g., adults aged 18–39 or those reporting cognitive disability). This approach ensures appropriate variance estimation by retaining the full sampling design, consistent with recommended survey analysis practices [26].

For categorical variables with more than two levels (age groups, race/ethnicity, education, income, employment status, smoking status, census region), we used ordered or multinomial logistic regression with year as the predictor. For binary outcomes (sex, insurance status, health behaviors, chronic conditions), we used logistic regression. We calculated and plotted predicted probabilities at years 2013 and 2024 using marginal effects, and computed percent change and *p*‐values for temporal trends. To formally compare temporal trends across 5‐year age subgroups within adults aged 18–39 years, we fit a survey‐weighted logistic regression model including year, age subgroup, and year × age subgroup interaction term, followed by pairwise comparisons between the 18–24 year‐old group and each older subgroup.

For chronic conditions assessed in alternating years (hypertension, hyperlipidemia), we included all available years in the regression models but report comparisons between 2013 and 2023. We estimated annual trends in the number of poor mental health days in the past 30 days using linear regression. For that analysis, we plotted predictive margins by year and cognitive disability status. Significance was determined at *p* < 0.05. All analyses were conducted using StataNow/MP 18.0 (StataCorp, College Station, TX).

### 2.6 Sensitivity Analyses

Because the current analysis includes an additional year of BRFSS data (2024) compared to our prior report [7], the first sensitivity analysis included all age groups (18–39, 40–54, 55–69, and 70+) to confirm that the 18–39 year old age group continues to have a trend of increasing self‐reported cognitive disability compared to other age groups. Using the same cohort but also excluding younger adults reporting any poor mental health days in a month, we report the trend in self‐reported cognitive disability from 2013–2024. The intent of this sensitivity analysis is to further isolate self‐reported cognitive impairment from the confounding of mood disorders. Finally, using the full range of age groups and no exclusions, we examined trends in depression prevalence and self‐reported cognitive disability from 2013–2024.

### 2.7 Exploratory Analyses

We performed three exploratory analyses using variables with limited availability. These analyses examined the prevalence of marijuana use, e‐cigarette use, and long COVID. Marijuana use was variably recorded in a subset of different states beginning in 2016 and never available in more than half of states. We transformed the monthly number of days of marijuana use into a binary variable: no marijuana vs. at least 1 day a month use. Current e‐cigarette use was measured beginning in 2016, but there were approximately 50% missing data in 2018, no available data in 2019, and near‐complete data for 2021–2024. Long COVID was assessed using variables from 2022 and 2023 and asked exclusively among respondents who reported prior COVID‐19 infection. The long COVID question was binary and open‐ended but phrased slightly differently in 2022 and 2023. In 2022 it was phrased “Did you have any symptoms lasting 3 months or longer that you did not have prior to having coronavirus or COVID‐19?” and in 2023 it was phrased “Do you currently have symptoms lasting 3 months or longer that you did not have prior to having coronavirus or COVID‐19?” [27, 28, 29]. Because these exploratory variables were not uniformly available across survey years or states, we report the unweighted year‐specific response counts for each variable in Table S2.

## 3 Results

### 3.1 Study Population and Baseline Characteristics

The primary analysis unweighted cohort included 812,557 adults aged 18–39 years without depression, of whom 49,905 (6.1%) reported a cognitive disability (Figure S1). The addition of 2024 BRFSS data continued to show an elevated prevalence of self‐reported cognitive disability among adults aged 18–39, consistent with the broader increase observed since 2013, although prevalence appeared to plateau between 2023 and 2024 (Figure S2A,B). Compared to younger adults without cognitive disability, individuals with cognitive disability were more likely to be in the youngest age stratum (35.3% vs. 25.9% aged 18–24 years, *p* < 0.001) and male (55.2% vs. 53.3%, p < 0.001). The racial and ethnic distribution differed significantly (*p* < 0.001), with higher representation of Hispanic (22.7% vs. 17.2%), Black (10.0% vs. 9.3%), and American Indian/Alaskan Native individuals (2.6% vs. 1.8%) (Table 1).

**TABLE 1:** Demographics after stratification by the primary outcome of self‐reported cognitive disability.

	No cognitive disability (*n* = 762,652)	Cognitive disability (*n* = 49,905)	*p*
Age group			
18–24	197,689 (25.9%)	17,598 (35.3%)	< 0.001
25–29	165,690 (21.7%)	10,922 (21.9%)	
30–34	190,620 (25.0%)	11,026 (22.1%)	
35–39	208,653 (27.4%)	10,359 (20.8%)	
Male Sex	406,253 (53.3%)	27,516 (55.2%)	< 0.001
Race/ethnicity			
White	460,872 (60.4%)	26,756 (53.6%)	< 0.001
Black	70,559 (9.3%)	4990 (10.0%)	
American Indian/Alaskan Native	14,016 (1.8%)	1306 (2.6%)	
Asian	41,786 (5.5%)	1492 (3.0%)	
Hispanic	130,891 (17.2%)	11,317 (22.7%)	
Other	44,507 (5.8%)	4043 (8.1%)	
Employment status			
Employed	569,779 (75.5%)	31,434 (63.8%)	< 0.001
Unemployed	44,811 (5.9%)	5614 (11.4%)	
Unable to Work	10,133 (1.3%)	3609 (7.3%)	
Student	82,008 (10.9%)	5813 (11.8%)	
Homemaker	47,230 (6.3%)	2593 (5.3%)	
Retired	1078 (0.1%)	190 (0.4%)	
Household income			
< $35,000	202,154 (31.1%)	19,533 (50.0%)	< 0.001
$35,000–$74,999	202,652 (31.2%)	10,856 (27.8%)	
$75,000+	245,622 (37.8%)	8690 (22.2%)	
Education level			
< High school	48,515 (6.4%)	6401 (12.9%)	< 0.001
High school or GED	201,383 (26.5%)	18,514 (37.3%)	
Some post high school	208,949 (27.5%)	14,529 (29.2%)	
College graduate	301,531 (39.7%)	10,249 (20.6%)	
Uninsured	99,933 (14.7%)	9186 (22.2%)	< 0.001
Hypertension	44,602 (10.8%)	4282 (16.5%)	< 0.001
Hyperlipidemia	37,354 (12.9%)	2925 (17.9%)	< 0.001
Diabetes Mellitus	13,789 (1.8%)	1499 (3.0%)	< 0.001
Chronic kidney disease	5428 (0.7%)	870 (1.7%)	< 0.001
Asthma	100,467 (13.2%)	10,751 (21.7%)	< 0.001
Myocardial infarction	3149 (0.4%)	611 (1.2%)	< 0.001
Stroke	2916 (0.4%)	790 (1.6%)	< 0.001
Obese	294,233 (41.8%)	18,822 (40.8%)	< 0.001
Current smoker	96,860 (12.9%)	11,225 (22.8%)	< 0.001
Current alcohol use	437,420 (59.2%)	26,737 (55.4%)	< 0.001
Physically inactive	129,291 (17.3%)	11,843 (24.2%)	< 0.001
Poor mental health days			
0 days/month	469,894 (62.3%)	15,568 (32.0%)	< 0.001
1–13 days/month	229,863 (30.5%)	18,066 (37.2%)	
14 or more days/month	54,712 (7.3%)	14,966 (30.8%)	
Census region			
Northeast	135,328 (17.7%)	8625 (17.3%)	< 0.001
Midwest	206,937 (27.1%)	12,181 (24.4%)	
South	207,107 (27.2%)	15,039 (30.1%)	
West	194,582 (25.5%)	12,401 (24.8%)	
Territories	18,698 (2.5%)	1659 (3.3%)	

Individuals with cognitive disability had higher unemployment (11.4% vs. 5.9%), inability to work (7.3% vs. 1.3%), and annual household income below $35,000 (50% vs. 31.1%) (all *p* < 0.001). The uninsured rate was higher among younger adults with cognitive disability (22.2% vs. 14.7%, *p* < 0.001). All measured chronic health conditions were significantly more prevalent among younger adults with cognitive disability, including hypertension (16.5% vs. 10.8%), hyperlipidemia (17.9% vs. 12.9%), diabetes mellitus (3.0% vs. 1.8%), chronic kidney disease (1.7% vs. 0.7%), asthma (21.7% vs. 13.2%), myocardial infarction (1.2% vs. 0.4%), and stroke (1.6% vs. 0.4%) (all *p* < 0.001). Obesity prevalence was similar between groups (40.8% vs. 41.8%).

Current smoking was nearly twice as common among younger adults with cognitive disability (22.8% vs. 12.9%, *p* < 0.001), and physical inactivity was higher (24.2% vs. 17.3%, *p* < 0.001). Rates of poor mental health were also elevated: 30.8% of individuals with cognitive disability reported ≥ 14 days/month versus 7.3% (*p* < 0.001). Geographic distribution also differed (*p* < 0.001), and younger adults with cognitive disability were more likely to reside in the South (30.1% vs. 27.2%) and less likely in the Midwest (24.4% vs. 27.1%).

### 3.2 Temporal Trends in Disability Prevalence

**FIGURE 1:**
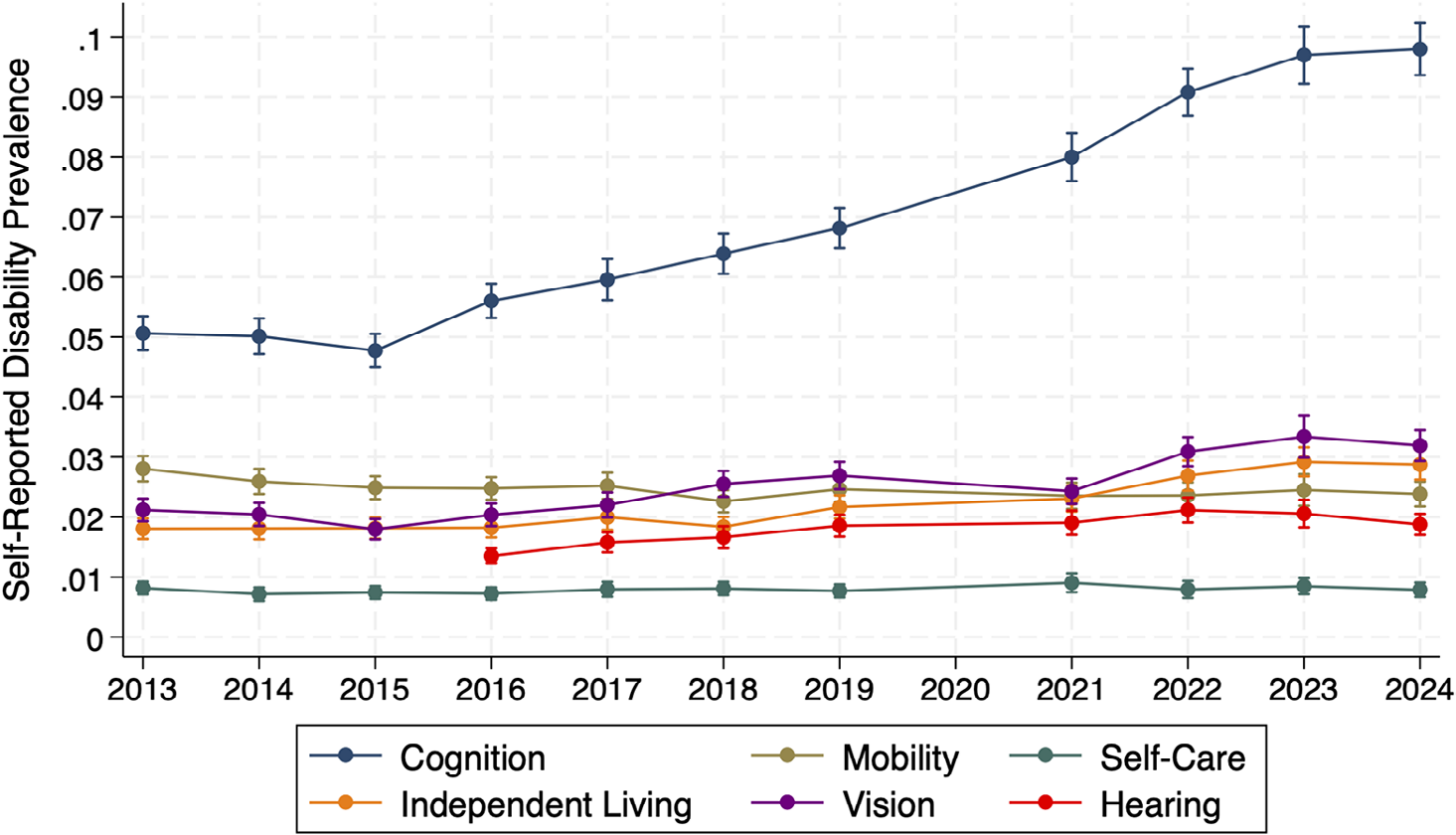
Trends in self‐reported disability prevalence by domain among U.S. adults aged 18–39 without depression, 2013–2024. Survey‐weighted prevalence estimates are shown for self‐reported cognitive, mobility, self‐care, independent living, vision, and hearing disability. Adults with a reported history of depression were excluded. Hearing disability data were available beginning in 2016. BRFSS 2020 data were excluded because of pandemic‐related data collection disruptions.

After weighting, the primary analytic cohort for the cognitive disability analysis represented an average of 71.3 million adults per year. Crude prevalence trends for the six disability types from 2013 to 2024 are seen in Figure 1 (see Figure S7 for individual disability types). The rate of cognitive disability rose from 5.1% in 2013 to 9.8% in 2024 (+93% relative), replicating and extending our prior findings. The increase first reached significance in 2016 (*p* = 0.009) and remained significant in subsequent years through 2024 (*p* < 0.001). Mobility disability showed a modest decline from 2.8% to 2.4% (−15%; multiple years *p* < 0.05). Self‐care disability was stable with no clear linear trend. Independent living disability increased from 1.8% to 2.9% (+59%; *p* < 0.001). Vision disability increased from 2.1% in 2013 to 3.2% in 2024 (+57%; *p* < 0.001). Hearing disability (available 2016–2024) increased from 1.4% to 1.9% (+39%; *p* < 0.001). These sensory‐related disabilities showed notable upward trends, paralleling those observed for cognitive disability.

**FIGURE 2:**
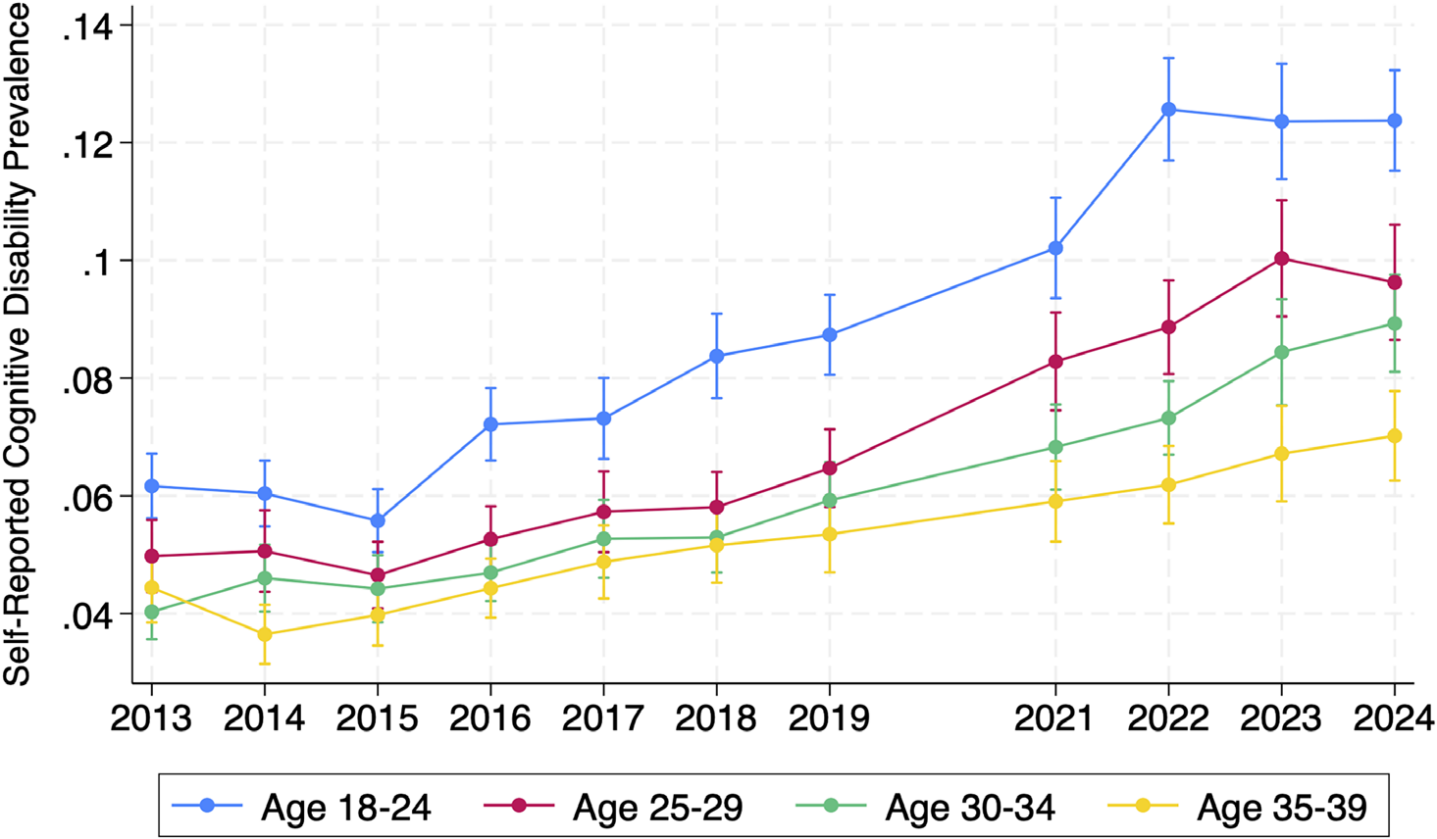
Trends in self‐reported cognitive disability prevalence by age group among U.S. adults aged 18–39 without depression, 2013–2024. Survey‐weighted annual prevalence estimates of self‐reported cognitive disability are shown across 5‐year age groups among U.S. adults aged 18–39 years without a reported history of depression. Age groups include 18–24, 25–29, 30–34, and 35–39 years. Points represent annual prevalence estimates, and error bars represent 95% confidence intervals. BRFSS 2020 data were excluded because of pandemic‐related data collection disruptions.

Figure 2 shows the pattern of self‐reported cognitive disability across 5‐year age bands within ages 18–39. Prevalence increased in every subgroup between 2013 and 2024, with the sharpest relative rise among 18–24‐year‐olds, whose rates nearly doubled from approximately 6.0% to 11.8%. Increases were also observed in the 25–29 (4.8% to 8.7%), 30–34 (4.9% to 8.4%), and 35–39 (4.5% to 7.7%) groups. Across all years, 18–24‐year‐olds consistently had the highest prevalence of self‐reported cognitive disability. In a survey‐weighted model including a year × age subgroup interaction, there was evidence that temporal trends differed across 5‐year age subgroups (interaction *p* = 0.024). Pairwise comparisons showed that the 18–24 years old group differed significantly from the 25–29, 30–34, and 35–39 groups.

### 3.3 Changes in Sociodemographic Factors Among Younger Adults With Cognitive Disability

Table 2 summarizes changes in sociodemographic and health characteristics between 2013 and 2024. Age distribution remained stable across the period (*p* = 0.795). The proportion of females decreased from 48.1% to 43.9% (−4.2%, *p* < 0.001). Racial and ethnic composition changed significantly (*p* = 0.026), with a decline in the proportion of White individuals (45.7% to 42.0%, −3.7%) and increases among Hispanic (29.0% to 31.6%, +2.6%), Asian (4.6% to 4.8%, +0.2%), and American Indian/Alaska Native participants (1.3% to 1.4%, +0.1%). Geographic distribution by census region also changed, but the shifts were very small over time.

**TABLE 2:** Changes from 2013 to 2024 in younger adults with self‐reported cognitive disability.

	Proportion in 2013	Proportion in 2024	Percentage point change 2013–2024	*p*
Age group				0.795
18–24	42.3%	41.9%	−0.4%	
25–29	20.5%	20.6%	+0.1%	
30–34	21.2%	21.4%	+0.2%	
35–39	15.9%	16.2%	+0.3%	
Female sex	48.1%	43.9%	−4.2%	< 0.001
Race/ethnicity				0.026
White	45.7%	42.0%	−3.7%	
Black	13.7%	13.7%	0%	
American Indian/Alaskan Native	1.3%	1.4%	+0.1%	
Asian	4.6%	4.8%	+0.2%	
Hispanic	29.0%	31.6%	+2.6%	
Other	5.7%	6.6%	+0.9%	
Employment status				< 0.001
Employed	55.6%	65.1%	+9.5%	
Unemployed	12.7%	11.2%	−1.5%	
Unable to work	7.9%	6.4%	−1.5%	
Student	17.5%	13.0%	−4.5%	
Homemaker	5.9%	4.0%	−1.9%	
Retired	0.4%	0.3%	−0.1%	
Household income				< 0.001
<$35,000	67.9%	37.5%	−30.4%	
$35,000–$74,999	19.8%	29.4%	+9.6%	
$75,000+	12.3%	33.1%	+20.8%	
Education level				< 0.001
< High school	22.9%	18.7%	−4.2%	
High school or GED	38.0%	35.9%	−2.1%	
Some post high school	27.9%	31.3%	+3.4%	
College graduate	11.3%	14.1%	+2.8%	
Uninsured	35.7%	19.7%	−16.0%	< 0.001
Hypertension^a^	17.9%	15.8%	−2.1%	0.180
Hyperlipidemia^a^	24.2%	21.7%	−2.5%	0.330
Diabetes Mellitus	4.3%	3.3%	−1.0%	0.204
Chronic kidney disease	1.6%	2.1%	+0.5%	0.377
Asthma	22.7%	19.4%	−3.3%	0.025
Myocardial infarction	1.6%	1.3%	−0.3%	0.618
Stroke	1.6%	1.3%	−0.3%	0.333
Obese	46.1%	41.3%	−4.8%	0.014
Current smoker	28.7%	14.1%	−14.6%	< 0.001
Current alcohol use	52.0%	52.7%	+0.7%	0.703
Physically inactive	26.9%	22.3%	−4.6%	< 0.001
Poor mental health days				< 0.001
0 days/month	36.9%	29.9%	−7.0%	
1–13 days/month	36.5%	36.9%	+0.4%	
14 or more days/month	26.6%	33.2%	+6.6%	
Census region				< 0.001
Northeast	14.8%	15.3%	+0.5%	
Midwest	18.2%	18.5%	+0.3%	
South	40.1%	40.0%	−0.1%	
West	25.1%	24.4%	−0.7%	
Territories	1.8%	1.7%	−0.1%	

^a^ For hypertension and hyperlipidemia, data are from 2023.

The proportion employed increased from 55.6% to 65.1% (+9.5%) and the proportion unable to work fell from 7.9% to 6.4% (−1.5%) (all *p* < 0.001). Income distributions shifted upward (*p* < 0.001) and the proportion with household income below $35,000 decreased from 67.9% to 37.5% (−30.4%). Educational attainment also improved (*p* < 0.001), with gains in college completion (11.3% to 14.1%, +2.8%) and some‐college education (27.9% to 31.3%, +3.4%). The proportion of uninsured adults decreased from 35.7% to 19.7% (−16.0%, *p* < 0.001).

Obesity declined from 46.1% to 41.3% (−4.8%, *p* = 0.014), and physical inactivity decreased from 26.9% to 22.3% (−4.6%, *p* < 0.001). Current smoking decreased from 28.7% to 14.1% (−14.6%, *p* < 0.001) and alcohol was stable at 52.0% to 52.7% (+0.7%, *p* = 0.703). Hypertension (17.9% to 15.8%, −2.1%, *p* = 0.180), hyperlipidemia (24.2% to 21.7%, −2.5%, *p* = 0.330), diabetes mellitus (4.3% to 3.3%, −1.0%, *p* = 0.204), and stroke (1.6% to 1.3%, −0.3%, *p* = 0.333) all decreased modestly, while chronic kidney disease showed a nonsignificant rise (1.6% to 2.1%, +0.5%, *p* = 0.377). Asthma also declined, from 22.7% to 19.4% (−3.3%, *p* = 0.025). Myocardial infarction remained rare and unchanged (1.6% to 1.3%, *p* = 0.618).

**FIGURE 3:**
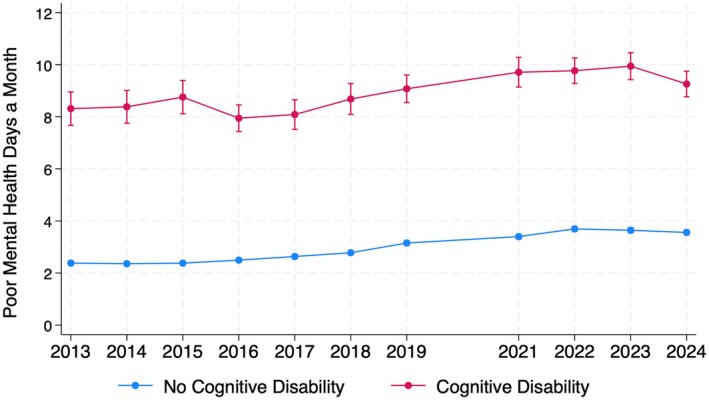
Poor mental health days per month by self‐reported cognitive disability status among U.S. adults aged 18–39, 2013–2024. Survey‐weighted predicted mean number of poor mental health days in the past 30 days is shown for younger adults with and without self‐reported cognitive disability. Poor mental health days were defined as days when mental health, including stress, depression, and problems with emotions, was not good. Markers represent predicted means, and error bars represent 95% confidence intervals. BRFSS 2020 data were excluded because of pandemic‐related data collection disruptions.

The number of days of poor mental health worsened significantly. The proportion reporting 14 or more days of poor mental health per month increased from 26.6% to 33.2% (+6.6%, *p* < 0.001). When treating the poor mental health variable as continuous (Figure 3), adults with cognitive disability reported substantially more poor mental health days than younger adults without (overall difference + 5.93 days in 2024, *p* < 0.001).

### 3.4 Sensitivity Analyses

The sensitivity analysis comparing the rate of cognitive disability in all age groups from 2013 to 2024 including younger adults with depression is shown in Figure S2, alongside the primary analysis that excludes them. The overall pattern was unchanged: cognitive disability increased significantly over time for the 18–39 age group (*p* < 0.001 for both). The adjusted prevalence of cognitive disability rose from 10% in 2013 to 17.8% in 2024, compared with 5.1% to 9.8% when younger adults with depression were excluded. The 18–39 age group still ultimately exceeded all others, reaching the highest rate of disability earlier—in 2018 when younger adults with depression were included, versus 2021 in the primary analysis.

When we further excluded younger adults with either depression or any poor mental‐health days (defined in BRFSS as days when “mental health was not good”), the observed rise persisted, although at lower absolute levels (Figure S3). In this analysis, the adjusted prevalence among 18–39‐year‐olds still rose from 2.8% in 2013 to 5.2% in 2024 (*p* < 0.001), at which point the 18–39 age strata again surpassed others, becoming the age group with the highest prevalence.

In the depression prevalence sensitivity analysis (Figure S4), the rate of depression prevalence increased substantially between 2013 and 2024 among adults aged 18–39 from 16.6% in 2013 to 25.3% in 2024 (*p* < 0.001). A similar upward trend was observed for ages 40–54 (19.7% to 22.5%), while ages 55–69 and 70+ trended downward or were stable (20.7% to 19.6% and 12.9% to 14.0%, respectively). The year‐by‐age interaction was significant (*p* < 0.001), reflecting a sharper and more sustained increase in depression among younger adults, mirroring the rates of cognitive disability seen in the primary analysis.

### 3.5 Exploratory Analyses

In exploratory analyses examining exposures with limited data, marijuana and e‐cigarette use both showed significant increases from 2016 to 2024. Unweighted year‐specific counts for these exploratory variables are provided in Table S2. Marijuana use at least 1 day a month rose steadily from 12.6% in 2016 to 19.4% in 2024 (*p* < 0.001), with a consistent twofold higher prevalence among younger adults reporting cognitive disability (Figure S5). Current e‐cigarette use increased from 6.3% in 2016 to 11.2% in 2024 (*p* < 0.001). Across all years, prevalence was higher among younger adults with cognitive disability compared to those without (20.2% vs. 9.9% in 2024), but the rate of increase over time did not differ significantly by self‐reported cognitive disability (Figure S6). Long COVID status was available only for 2022–2023 and the prevalence declined from 7.0% in 2022 to 5.5% in 2023, when it was two‐fold higher among younger adults with cognitive disability (9.8% vs. 4.9%, *p* = 0.045).

## 4 Discussion

With the inclusion of 2024 BRFSS data, self‐reported cognitive disability among U.S. adults aged 18–39 remained substantially higher than in 2013, with the upward trend beginning before the COVID‐19 pandemic and remaining elevated in subsequent years [3, 7, 30]. When excluding respondents with depression, prevalence increased from 5.1% in 2013 to 9.8% in 2024. When including respondents with depression, prevalence rose from 10.0% to 17.8%, representing 16,174,264 U.S. adults aged 18–39 in 2024.

Importantly, these findings should not be interpreted as evidence of a definitive increase in neurologic disease among younger adults. The BRFSS cognitive disability item reflects self‐reported serious difficulty concentrating, remembering, or making decisions, which may represent subjective cognitive symptoms, functional cognitive difficulty, psychiatric or stress‐related symptoms, neurodevelopmental factors, medical or sleep‐related contributors, or changing patterns of symptom recognition and disclosure.

The highest rates of cognitive disability were in 18–24‐year‐olds, but the rise was significant in all 5‐year strata within the 18–39 age group. Although some individuals in the earlier years of the analysis would have aged into older categories by the end of the study period, the continued increase in all 5‐year strata indicates a generation‐level phenomenon that may persist across the adult life course. In contrast, other disability domains showed mixed trajectories: independent living, hearing, and vision disabilities increased, while mobility and self‐care disabilities were stable or declined.

The current study focuses on adults aged 18–39 because prior work demonstrated that this group was the primary driver of the national increase in self‐reported cognitive disability. However, the contrast with older adults remains important: the relative stability or decline in older groups suggests that the rise among younger adults is unlikely to reflect a uniform survey artifact and may instead reflect generational, behavioral, psychiatric, social, or reporting‐related factors.

Because all six disability questions appear in the same BRFSS section, this selective pattern argues against a uniform reporting or methodological artifact [2, 4, 31]. Notably, the increases in vision (57%) and hearing (39%) disability among younger adults are significant. Both are modifiable risk factors for cognitive impairment, with hearing loss in midlife and vision loss in late life estimated to account for approximately 7% and 2% of dementia risk, respectively [32]. Rising sensory disability in this age group may signal future cognitive vulnerability.

At the same time, socioeconomic and health behavior indicators among younger adults with cognitive disability improved. Between 2013 and 2024, employment rose 17% [33, 34], uninsured rates fell by almost half, college completion increased, and nominal household income shifted upward. Current smoking halved [35, 36, 37] and physical inactivity declined [38, 39]. These improvements parallel broader public‐health trends, such as the Affordable Care Act, and demonstrate that the growing group of young adults reporting cognitive disability is not simply composed of younger adults with worsening economic or medical disadvantage [40, 41].

In contrast, the mental health burden worsened steadily [42]. Even after excluding respondents with depression, the proportion of individuals with cognitive disability reporting at least 14 poor mental‐health days per month increased by one‐quarter and poor mental health days per month was 3.6 versus 9.3 for younger adults without versus with cognitive disability in 2024. We also found that depression prevalence increased most sharply among younger adults [43], suggesting that both population‐level depression and subclinical mood symptoms are rising in this generation and may be an important driver of the rise in self‐reported cognitive disability [12, 44].

The relationship between poor mental health and self‐reported cognitive difficulty is biologically plausible, although it should not be interpreted as causal in this cross‐sectional survey. The prefrontal cortex supports higher‐order executive functions, including attention regulation, working memory, cognitive flexibility, and decision‐making. Experimental and translational studies indicate that acute and chronic stress can disrupt prefrontal cortical network function through catecholaminergic and glucocorticoid signaling pathways, shifting control away from top‐down executive systems toward more reactive or habitual responses [45, 46]. These stress‐related effects on executive function are relevant to the BRFSS cognitive‐disability item, which asks about serious difficulty concentrating, remembering, or making decisions. In parallel, depression and psychological distress are associated with deficits in attention, memory, and executive function [19, 20]. Therefore, the parallel rise in poor mental health and self‐reported cognitive disability among younger adults may reflect, at least in part, stress‐ and mood‐related cognitive symptoms, although BRFSS cannot determine whether these symptoms represent objective cognitive impairment, psychiatric burden, functional difficulty, or changing patterns of symptom recognition and reporting.

Furthermore, while every generation experiences mental health challenges, the recent period combined distinctive social and technological changes that may be particularly relevant to younger adults. Although not directly measured in BRFSS, increasing digital and social media exposure, media multitasking, and constant notification‐driven attentional demands may contribute to perceived difficulty with concentration and decision‐making [14, 47, 51]. This hypothesis is consistent with the age‐specific pattern observed in this study, particularly the high prevalence among adults aged 18–24, and may help explain why older age groups were comparatively stable or declining. At the same time, the observed trend is likely multifactorial, with potential contributions from pandemic‐related disruption, social isolation, long‐COVID sequelae, and escalating psychosocial stressors [43, 44, 49, 50, 52, 53].

Additionally, because excluding younger adults with either depression or any poor mental health days a month did not materially change the age‐related pattern (Figure S3), additional explanations merit consideration. The higher prevalence of marijuana and e‐cigarette use among younger adults with self‐reported cognitive disability suggests that substance‐use patterns may contribute to perceived or functional difficulty in this population [54]. Although long COVID was also more common among younger adults with self‐reported cognitive disability in 2022–2023, it is unlikely to explain the overall rise because the upward trend began before the COVID‐19 pandemic [55]. Therefore, long COVID should be interpreted as one possible contributor after 2020 rather than the primary driver of the observed 2013–2024 trend. Recent BRFSS analyses using the Adverse Childhood Experience (ACE) module also illustrated that early‐life adversity is associated with subjective cognitive impairment, specifically among younger adults [56].

Finally, reduced stigma and greater recognition of neurodiversity (e.g., adult diagnoses of ADHD or autism) may increase self‐identification and make younger adults more willing to acknowledge cognitive difficulties [57, 58, 59]. These shifts in recognition and disclosure could increase self‐reported cognitive disability even without a parallel increase in objectively measured neurologic disease and may explain why individuals who report cognitive disability today appear healthier and more socioeconomically successful than in 2013.

Taken together, these data portray a generation navigating unprecedented cognitive challenges. Despite improvements in education, employment, and insurance, younger adults increasingly perceive cognitive disability. This underscores the need for integrative strategies: continued surveillance linking cognitive and mental‐health indicators, expanded access to affordable, age‐appropriate mental‐health care, and preventive efforts that reduce stress exposure and promote cognitive resilience through improved sleep, more physical activity, and healthier technology engagement.

### 4.1 Strengths and Limitations

Strengths of this study include a large, population‐based sample, consistent methodology, comprehensive domain coverage, and appropriate statistical methods, including subpopulation analysis and exclusion of participants with depression to isolate cognitive disability. However, individuals with anxiety disorders (e.g., panic disorders, generalized anxiety disorder, PTSD, or OCD) were not excluded, as the BRFSS does not identify these separately and such conditions can also cause cognitive symptoms [60].

Limitations include reliance on self‐reported data, which are subject to bias and evolving reporting patterns, a single‐question assessment that does not capture severity or etiology, exclusion of 2020 data limiting assessment of pandemic effects, and selected‐year measurement of covariates. Long COVID analyses were exploratory and should be interpreted cautiously because the variable was available only in 2022–2023, was limited to respondents with prior COVID‐19 infection, relied on self‐report, and used slightly different question wording across years. Importantly, cognitive disability in this study reflects self‐reported serious difficulty with concentration, remembering, or decision‐making, not clinically diagnosed cognitive impairment or dementia. This outcome likely captures a heterogeneous construct that may include subjective cognitive complaints, psychiatric symptoms, sleep disorders, medication effects, substance use beyond the limited measures available in BRFSS, traumatic brain injury or repetitive head‐impact exposure, neurodevelopmental conditions such as ADHD or autism, and other unmeasured contributors. BRFSS does not provide sufficient clinical detail to distinguish among these possible etiologies, assess symptom severity, or determine whether reported difficulties reflect objective neurocognitive impairment versus perceived or functional cognitive difficulty. While our analyses argue against a simple survey artifact, measurement limitations warrant acknowledgment. The cognitive‐disability question is inherently subjective and may reflect both genuine neurocognitive symptoms and shifting self‐perception.

Changing societal attitudes around neurodiversity and the normalization of attentional or executive difficulties (e.g., increased public identification with ADHD or autism) may also influence self‐reporting, potentially lowering the threshold for perceiving or disclosing cognitive problems even without corresponding changes in objective function. Because BRFSS is a U.S.‐based survey, these findings should be interpreted within the context of U.S.‐specific social, cultural, healthcare, and policy factors. Patterns of self‐reported cognitive disability may differ in countries with different healthcare systems, disability classifications, cultural norms around mental health and cognitive symptoms, and access to diagnostic or support services. Therefore, the results may not directly generalize to non‐U.S. populations. However, similar trends could plausibly emerge in other settings experiencing comparable generational changes in mental health burden, digital, and social media exposure, and evolving recognition of neurodiversity. Future work should combine BRFSS data with objective cognitive testing and biomarker cohorts, explore within‐person trajectories, and investigate synergistic mechanisms that could explain this persistent rise.

## 5 Conclusion

From 2013–2024, self‐reported cognitive disability among U.S. adults aged 18–39 nearly doubled despite improvements in socioeconomic and health metrics. This rise highlights a growing burden of perceived or functional cognitive difficulty among younger adults and underscores the need to better understand its neurologic, psychiatric, behavioral, and social contributors.

## Author Contributions

A.H. and K.‐H.W. contributed to the conception and design of the study. A.H. performed the data analysis. A.H., M.M., and K.‐H.W. contributed to drafting a significant portion of the manuscript. A.H., M.M., and K.‐H.W. contributed to manuscript revision and editing. C.D.A., A.F., A.A., A.F.‐Z., J.C.M., M.L.A., and K.N.S. provided critical intellectual input through review and revision of multiple manuscript versions. All authors reviewed and approved the final manuscript.

## Funding

This work was supported by National Institute of Neurological Disorders and Stroke funding (UG3NS133209, UH3NS130228, R01NS130189, R21NS138995). Dr. Sheth reports funding by National Institute of Neurological Disorders and Stroke U01NS106513, R01NS11072, R01NR018335, R01EB301114, R01MD016178, R03NS112859, U24NS107215, U24NS107136, and American Heart Association 17CSA33550004. Dr. Anderson reports funding by U01NS069673, RF1NS139183, 21SFRN812095, and the MGB Department of Neurology.

## Conflicts of Interest

A.H. has received consulting fees from Integra and Novo Nordisk, royalty fees from UpToDate, and holds equity in TitinKM and Certus. K.N.S. reports compensation from Sense and Zoll for data and safety monitoring services; consulting fees from Cerevasc, Rhaeos, and Certus; and a patent pending for stroke wearable technologies licensed to Alva Health. C.D.A. has received sponsored research support from Bayer AG and has consulted for MPM BioImpact and ApoPharma; these relationships are unrelated to the work presented. J.C.M. consults or has consulted with Customer Value Partners, BridgeBio, Determined Health, Apple, Neumarker, and BlackThorn Therapeutics; has received research funding from Janssen Research and Development; serves on the scientific advisory boards of Pastorus and Modern Clinics; and receives royalties from Guilford Press, Lambert, Oxford University Press, and Springer. The remaining authors declare no conflicts of interest.

## Supporting information


**Figure S1:** Derivation of the unweighted cohort.
**Figure S2:** Self‐reported cognitive disability prevalence by age group, 2013–2024, including and excluding adults with depression.
**Figure S3:** Self‐reported cognitive disability prevalence by age group after excluding adults with depression or any poor mental health days, 2013–2024.
**Figure S4:** Rate of depression prevalence by age group among U.S. adults, 2013–2024.
**Figure S5:** Rate of one or more days of marijuana use per month in younger adults with versus without self‐reported cognitive disability from 2016–2024.
**Figure S6:** Rate of current e‐cigarette use in younger adults with versus without self‐reported cognitive disability from 2016–2024 (excluding 2019).
**Figure S7:** Age‐adjusted prevalence of the six BRFSS disability domains among U.S. adults, 2013–2024.
**Table S1:** BRFSS self‐reported disability questions.
**Table S2:** Unweighted available response counts for exploratory variables by survey year.

## Data Availability

The data that support the findings of this study are publicly available through the Centers for Disease Control and Prevention Behavioral Risk Factor Surveillance System (BRFSS): https://www.cdc.gov/brfss/annual_data/annual_data.htm.
