# Supplementary material for: Factors Associated With the Rising Trend in Self‐Reported Cognitive Disability Among U.S. Adults Aged 18–39 From 2013–2024
Source: Ann Clin Transl Neurol. 2026 Jul 5:10.1002/acn3.70464. Online ahead of print. doi: 10.1002/acn3.70464 (PMC13395017; doi:10.1002/acn3.70464)
Supplement: Supplementary file 1 — Figure S1: Derivation of the unweighted cohort. Figure S2: Self‐reported cognitive disability prevalence by age group, 2013–2024, including and excluding adults with depression. Figure S3: Self‐reported cognitive disability prevalence by age group after excluding adults with depression or any poor mental health days, 2013–2024. Figure S4: Rate of depression prevalence by age group among U.S. adults, 2013–2024. Figure S5: Rate of one or more days of marijuana use per month in younger adults with versus without self‐reported cognitive disability from 2016–2024. Figure S6: Rate of current e‐cigarette use in younger adults with versus without self‐reported cognitive disability from 2016–2024 (excluding 2019). Figure S7: Age‐adjusted prevalence of the six BRFSS disability domains among U.S. adults, 2013–2024. Table S1: BRFSS self‐reported disability questions. Table S2: Unweighted available response counts for exploratory variables by survey year. [file ACN3-9999-0-s001.docx]

**SUPPLEMENTARY MATERIAL**

**Supplementary Table 1.** *BRFSS self-reported disability questions.*

| **Types of Disability** | **Related Questions in BRFSS** |
| --- | --- |
| Cognitive disability | Because of a physical, mental, or emotional condition, do you have serious difficulty concentrating, remembering, or making decisions? |
| Mobility disability | Do you have serious difficulty walking or climbing stairs? |
| Hearing disability | Are you deaf or do you have serious difficulty hearing? |
| Vision disability | Are you blind or do you have serious difficulty seeing, even when wearing glasses? |
| Independent living disability | Because of a physical, mental, or emotional condition, do you have difficulty doing errands alone such as visiting a doctor's office or shopping? |
| Self-care disability | Do you have difficulty dressing or bathing? |

**Supplementary Table 2.** *Unweighted available response counts for exploratory variables by survey year*

| **Survey year** | **Analytic cohort, N** | **Marijuana use available, n (%)** | **E-cigarette use available, n (%)** | **Long COVID available, n (%)** |
| --- | --- | --- | --- | --- |
| 2016 | 83,404 | 17,013 (20.40) | 79,270 (95.04) | 0 (0.00) |
| 2017 | 78,052 | 10,630 (13.62) | 73,627 (94.33) | 0 (0.00) |
| 2018 | 77,433 | 17,852 (23.05) | 45,333 (58.54) | 0 (0.00) |
| 2019 | 72,467 | 14,722 (20.32) | 0 (0.00) | 0 (0.00) |
| 2021 | 76,449 | 21,246 (27.79) | 71,676 (93.76) | 0 (0.00) |
| 2022 | 75,884 | 14,300 (18.95) | 69,300 (91.32) | 65,840 (86.76) |
| 2023 | 73,190 | 13,139 (17.95) | 68,522 (93.62) | 65,938 (90.09) |
| 2024 | 79,292 | 20,349 (25.66) | 73,306 (92.45) | 0 (0.00) |
| **Total** | **616,171** | **129,331 (20.99)** | **481,034 (78.07)** | **131,778 (21.39)** |

***** *Counts are unweighted. Percentages reflect the proportion of the analytic cohort in each survey year with an available response for the corresponding exploratory variable. BRFSS data from 2020 were excluded. Long COVID was assessed only in 2022 and 2023 among respondents who reported prior COVID-19 infection. E-cigarette use was not available in 2019. Marijuana use was available only in a subset of states across survey years.*

**Supplementary Figure 1.** *Derivation of the unweighted cohort.*

**Supplementary Figure 2*.*** *Self-reported cognitive disability prevalence by age group, 2013–2024, including and excluding adults with depression.*
***A. Analysis including adults with depression*

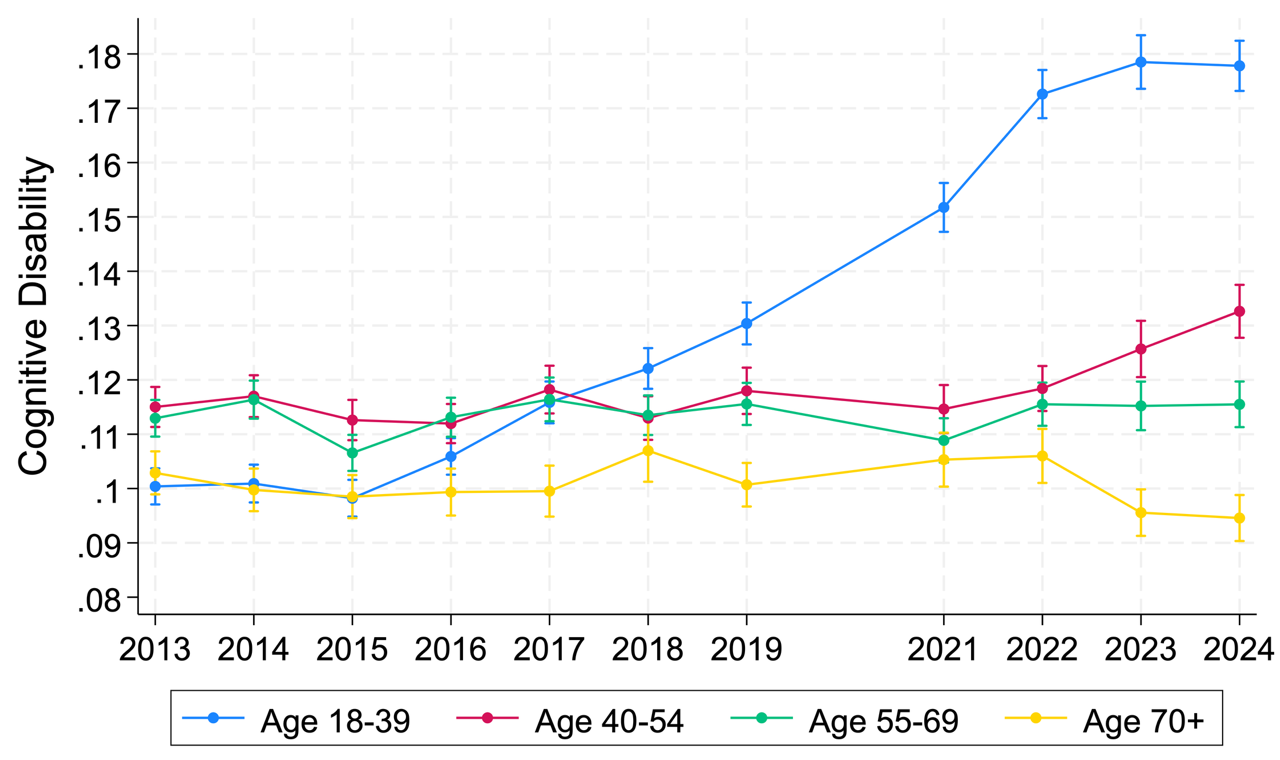
**

***B. Primary analysis excluding adults with depression***

**
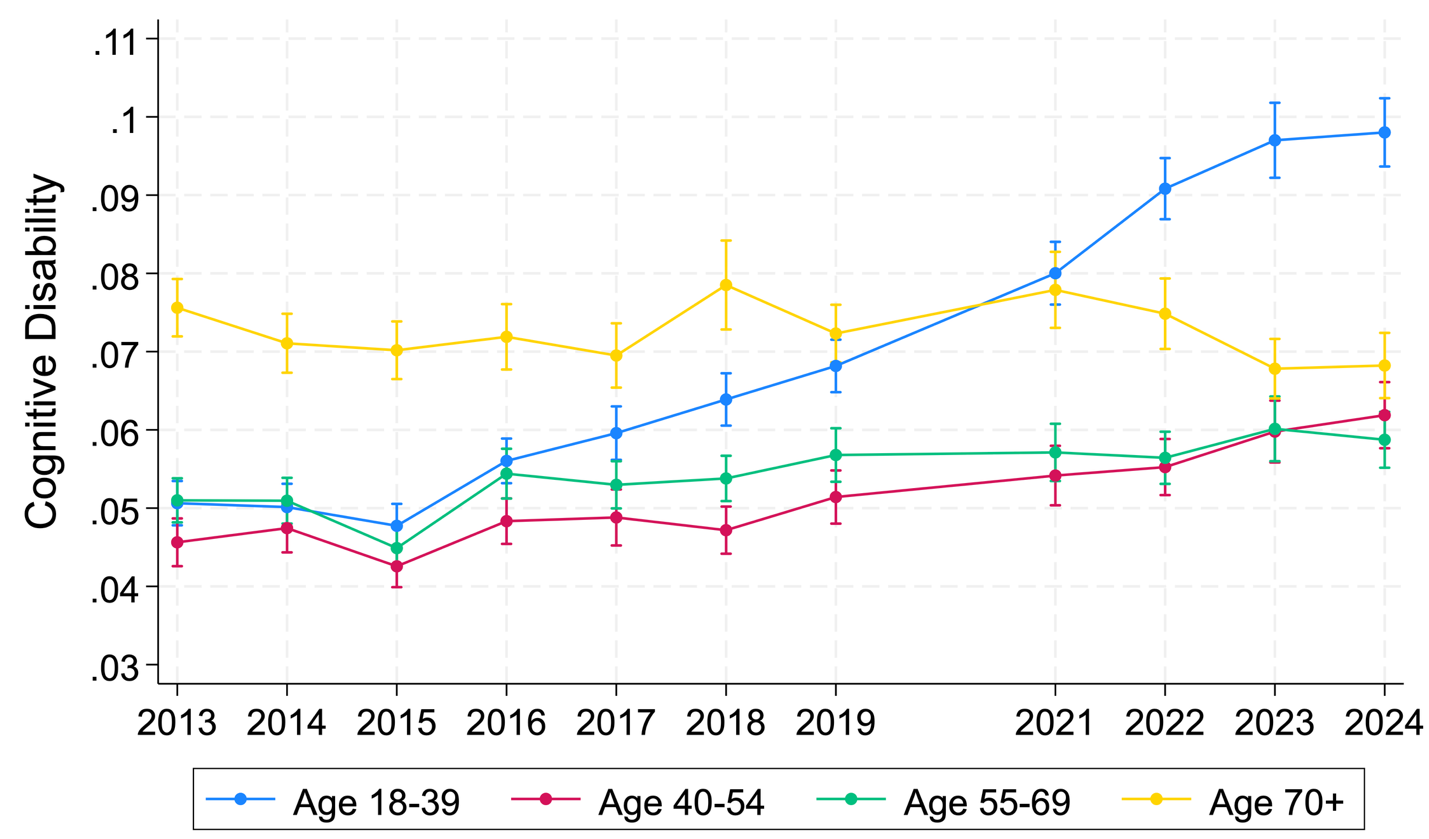
**

*Survey-weighted prevalence estimates of self-reported cognitive disability are shown by age group among U.S. adults in the BRFSS.* ***Panel A*** *includes adults with and without a history of depression.* ***Panel B*** *shows the primary analysis excluding adults who reported ever being told they had a depressive disorder. Points represent annual prevalence estimates, and error bars represent 95% confidence intervals. BRFSS 2020 data were excluded because of pandemic-related data collection disruptions.*

**Supplementary Figure 3.** *Self-reported cognitive disability prevalence by age group after excluding adults with depression or any poor mental health days, 2013–2024.*
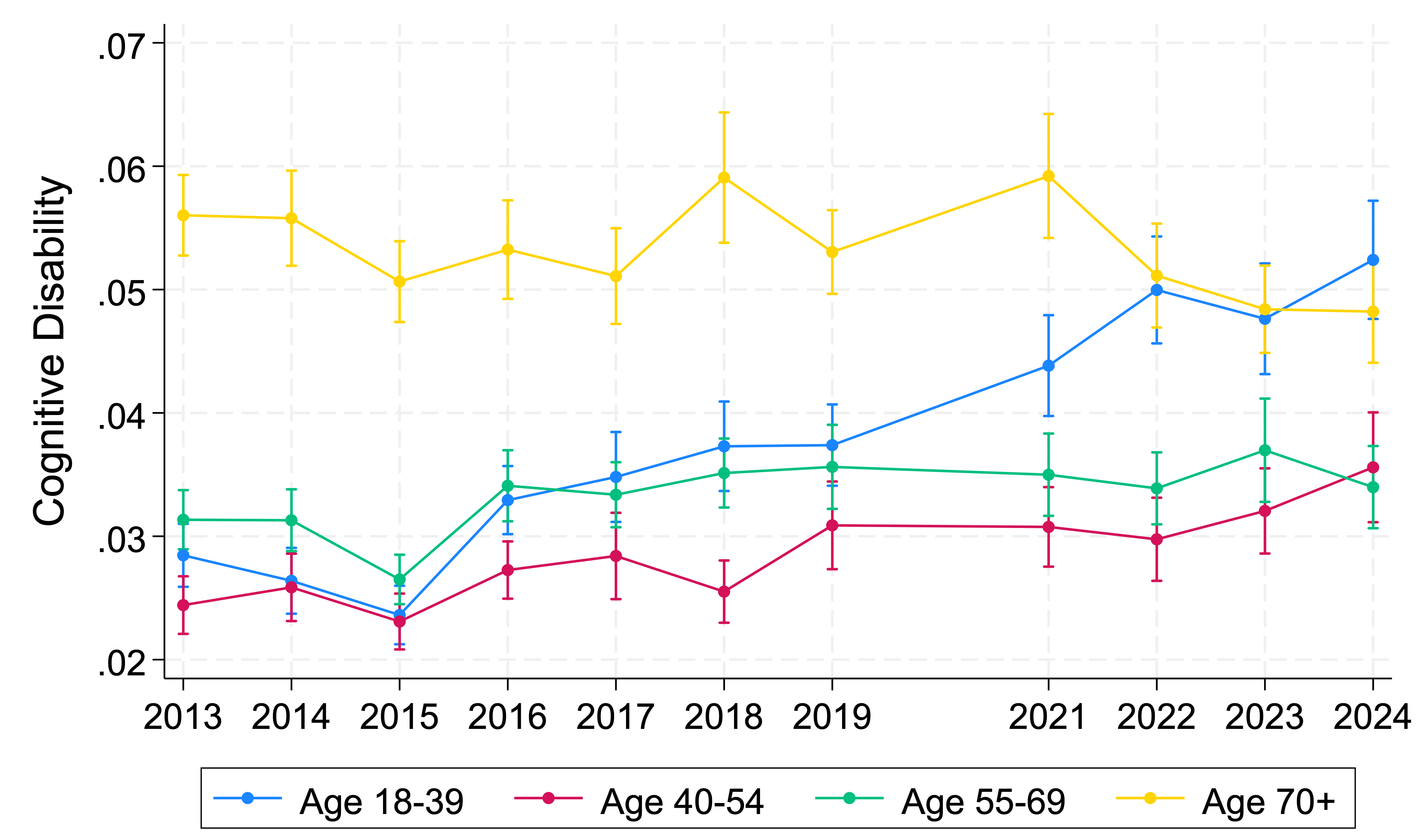


*Survey-weighted prevalence estimates of self-reported cognitive disability are shown by age group among U.S. adults in the BRFSS after excluding respondents who reported a history of depression or any poor mental health days in the past 30 days. Points represent annual prevalence estimates, and error bars represent 95% confidence intervals. BRFSS 2020 data were excluded because of pandemic-related data collection disruptions.*

**Supplementary Figure 4**. *Rate of depression prevalence by age group among U.S. adults, 2013–2024.*


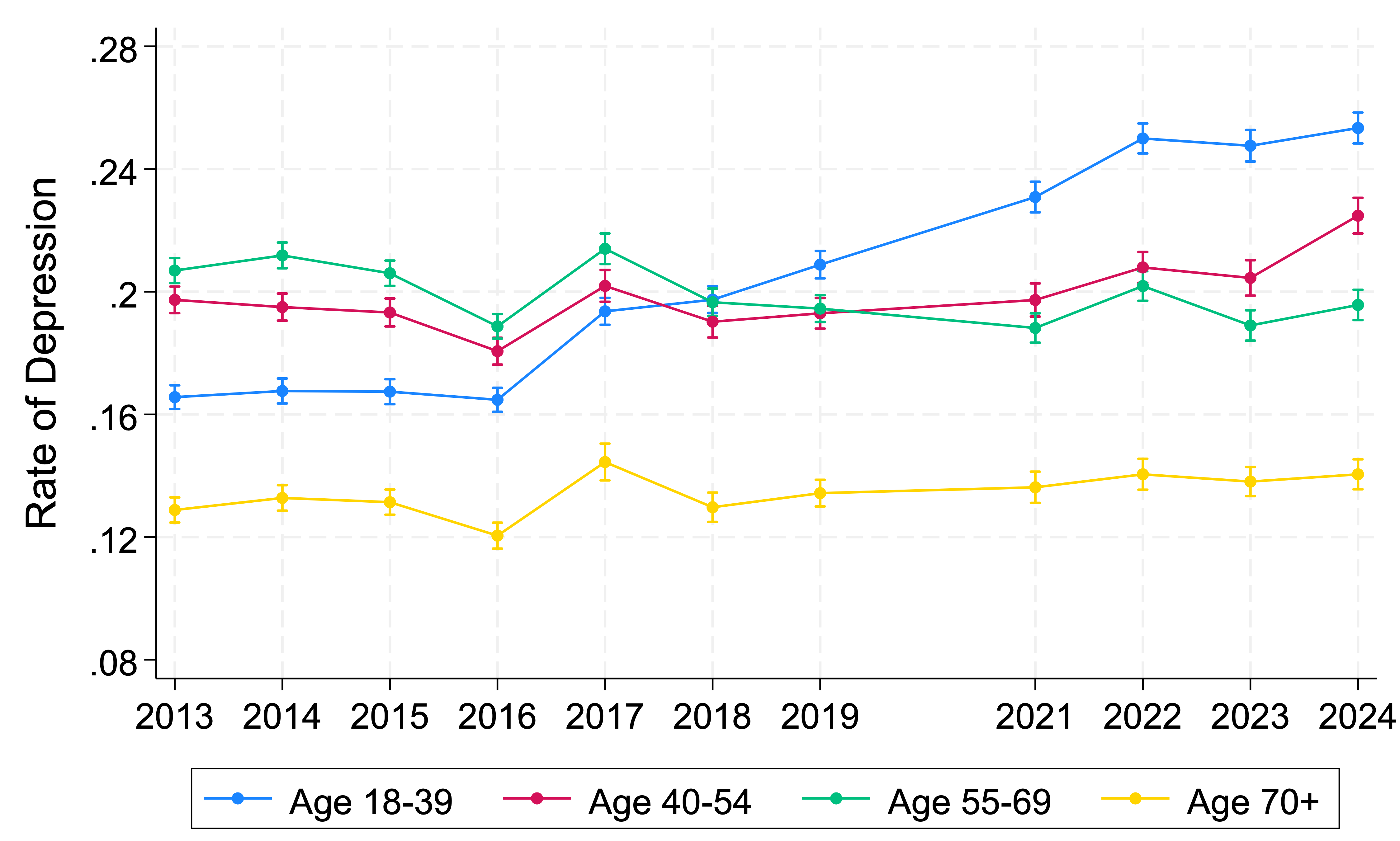


*Survey-weighted prevalence estimates of reported history of depression are shown by age group among U.S. adults in the BRFSS. Depression was defined as ever being told by a health professional that the respondent had a depressive disorder, including depression, major depression, dysthymia, or minor depression. Points represent annual prevalence estimates, and error bars represent 95% confidence intervals. BRFSS 2020 data were excluded because of pandemic-related data collection disruptions.*

**Supplementary Figure 5.** *Rate of one or more days of marijuana use per month in younger adults with versus without self-reported cognitive disability from 2016-2024.*

**
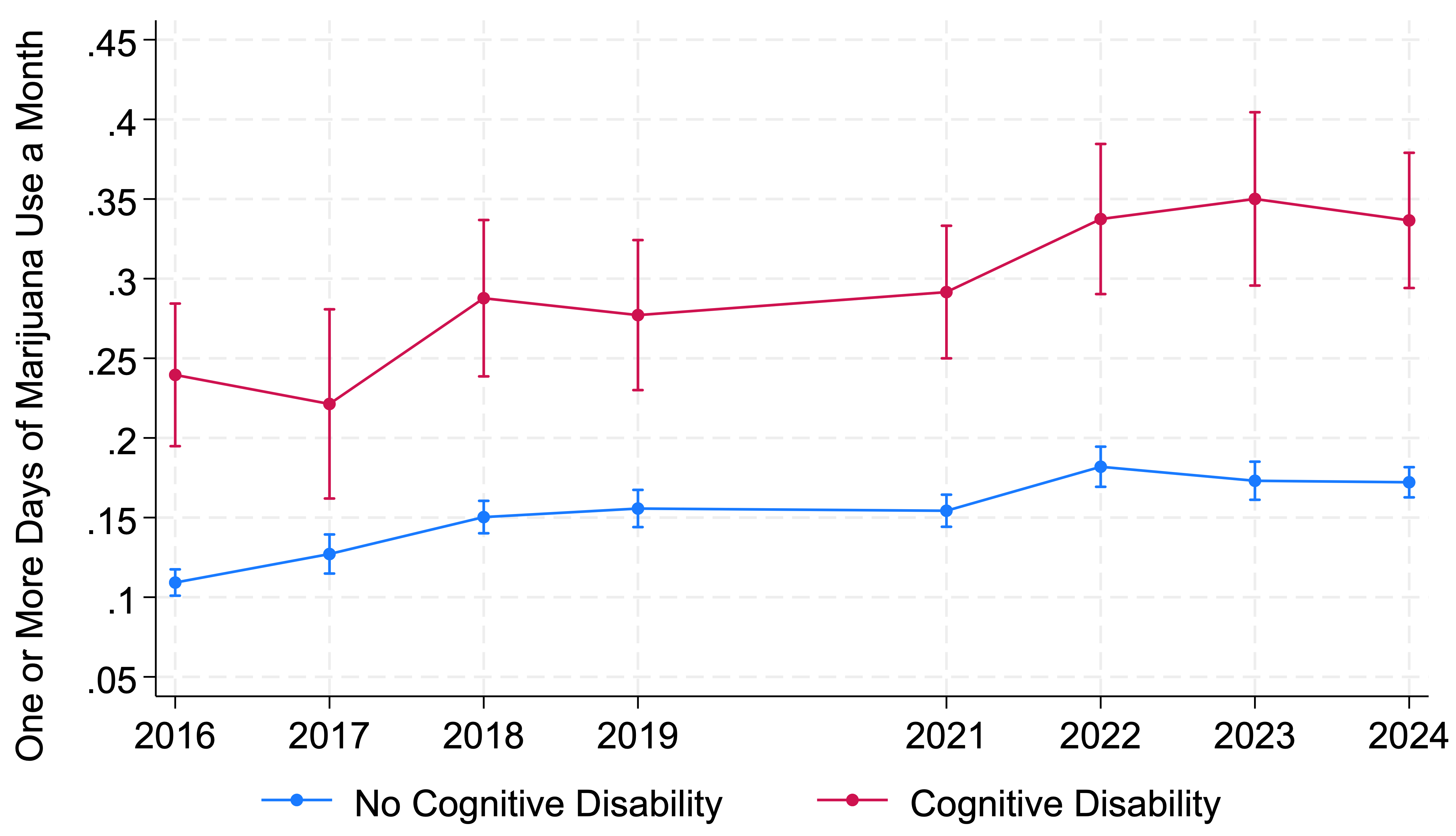
**

*Survey-weighted prevalence estimates are shown for U.S. adults aged 18–39 years in the BRFSS, stratified by self-reported cognitive disability status. Marijuana use was defined as use on 1 or more days in the past 30 days. Data was available only in a subset of states by year. Points represent annual prevalence estimates, and error bars represent 95% confidence intervals. BRFSS 2020 data were excluded.*

**Supplementary Figure 6.** Rate of current e-cigarette use in younger adults with versus without self-reported cognitive disability from 2016-2024 (excluding 2019).


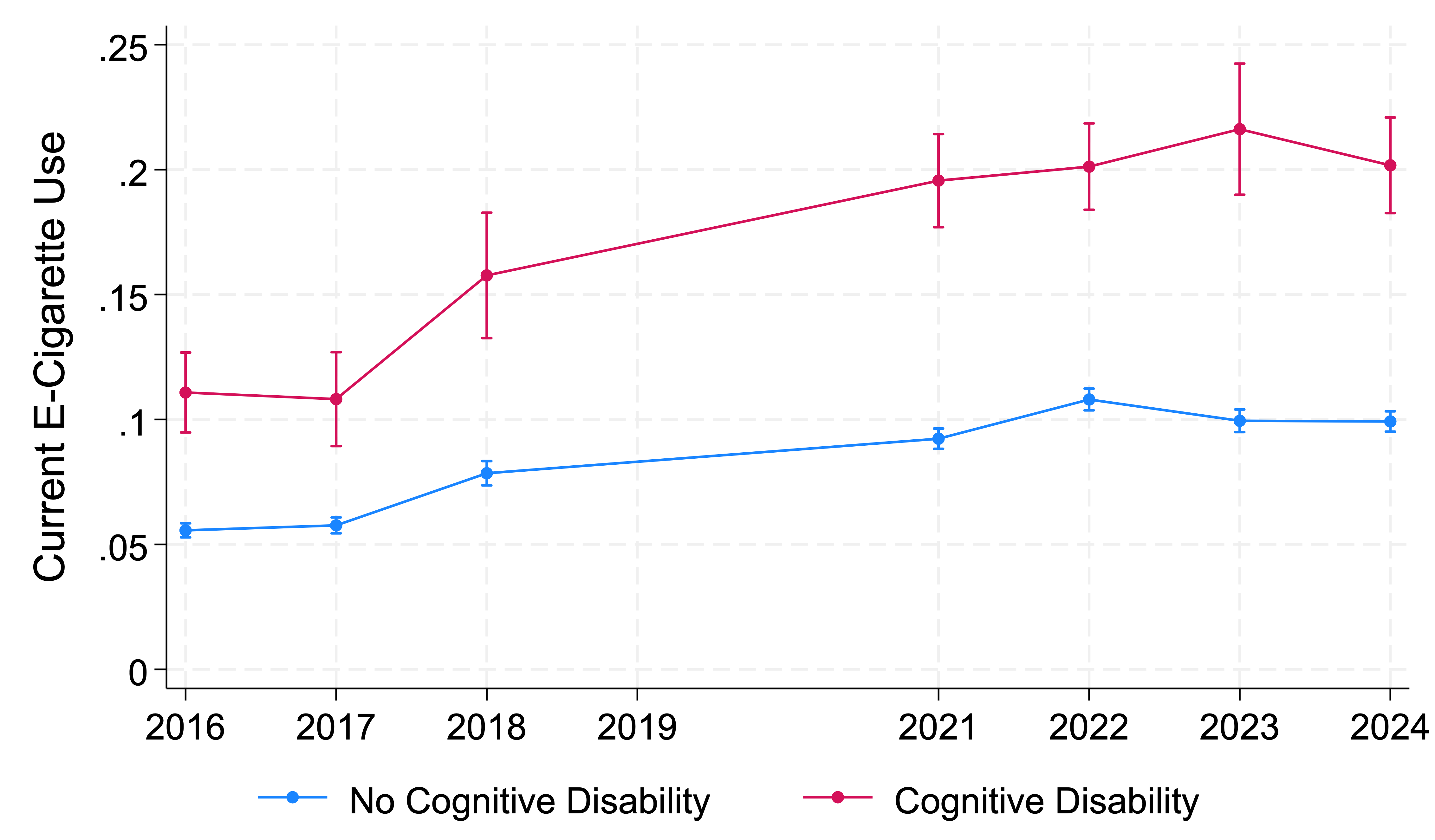


*Survey-weighted prevalence estimates of current e-cigarette use are shown for U.S. adults aged 18–39 years in the BRFSS, stratified by self-reported cognitive disability status. Data are shown for available years beginning in 2016; 2019 data were unavailable, and BRFSS 2020 data were excluded because of pandemic-related data collection disruptions. Points represent annual prevalence estimates, and error bars represent 95% confidence intervals.*

**Supplementary Figure 7:** *Age-adjusted prevalence of the six BRFSS disability domains among U.S. adults, 2013–2024.*


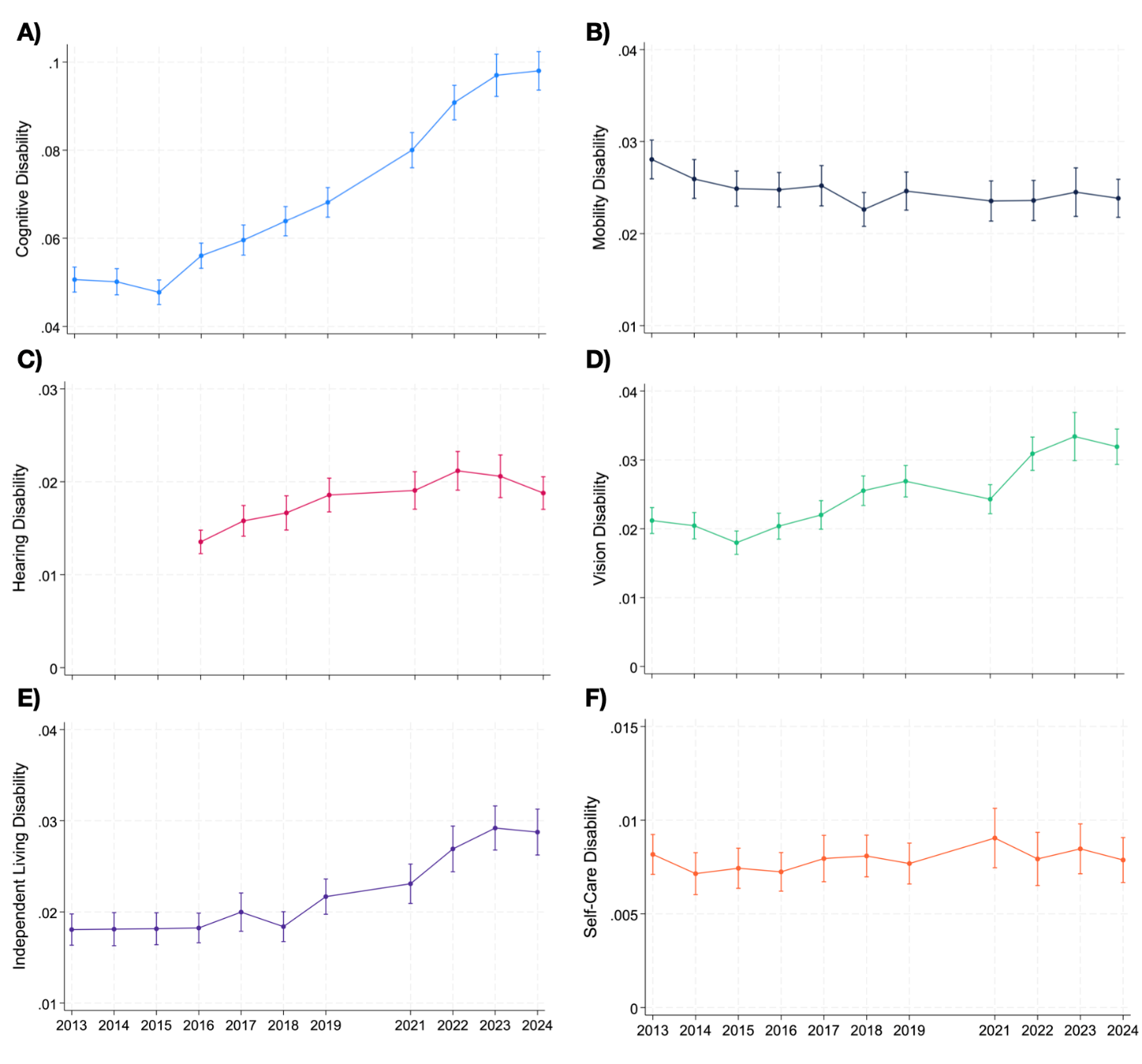


*Age-adjusted, survey-weighted prevalence estimates are shown for the six disability domains measured in the BRFSS: (A) self-reported cognitive disability, (B) mobility disability, (C) hearing disability, (D) vision disability, (E) independent living disability, and (F) self-care disability. Points represent annual prevalence estimates, and error bars represent 95% confidence intervals. BRFSS 2020 data were excluded because of pandemic-related data collection disruptions. Hearing disability data were available for 2016–2024 only.*
